# Effects of physically active lessons and active breaks on cognitive performance and health indicators in elementary school children: a cluster randomized trial

**DOI:** 10.1186/s12966-025-01789-6

**Published:** 2025-07-09

**Authors:** João Carlos N. Melo, Julian Tejada, Ellen Caroline M. Silva, José Ywgne, David N. Oliveira, Larissa Gandarela, Danilo R. Silva

**Affiliations:** 1 Graduate Program in Health Sciences, Universidade Estadual de Londrina (UEL), Londrina, PR Brazil; 2 Department of Phychology, Universidade Federal de Sergipe (UFS), São Cristóvão, SE Brazil; 3 Graduate Program in Movement Sciences, Universidade Federal de Sergipe (UFS), São Cristóvão, SE Brazil; 4 Department of Physical Education, Universidade Federal de Sergipe (UFS), São Cristóvão, SE Brazil; 5 Graduate Program in Health Sciences, Universidade Federal de Sergipe (UFS), Aracaju, Brazil; 6 Faculty of Health Sciences, Universidad Autónoma de Chile, Providencia, Chile; 7 Bradford Institute for Health Research, Bradford Teaching Hospitals NHS Foundation Trust, Bradford, UK

**Keywords:** Physically Active Learning, Executive Function, Health Indicator, Classroom Physical Activity

## Abstract

**Background:**

This cluster‐randomized trial examined the effects of active breaks (AB) and physically active lessons (PAL) on cognitive function and health indicators in elementary school children.

**Methods:**

Six schools were randomly assigned to three groups: AB group (n = 61), PAL group (n = 77), and a control group (CTL, n = 46). First-year elementary school students participated (6.9 ± 0.6 years; 52.7% girls), and the interventions lasted eight weeks. Cognitive function was measured via reaction time and correct responses on computerized tests (Go/NoGo, DigitSpan, Mental Rotation, Visual Search, and Cueing Posner). Secondary outcomes included physical activity, quality of life, daytime sleepiness, and school perception.

**Results:**

Significant group-by-time interactions were found in four tests: Go/NoGo (reaction time: *p* = 0.045), DigitSpan (correct responses: *p* = 0.020), Mental Rotation (reaction time: *p* = 0.049), and Cueing Posner (reaction time: *p* = 0.017). Only the PAL group presented a reduction in reaction time in inhibitory control (Go/NoGo) (change from baseline [Δ] = -106.4 ms; *p* < 0.001; d = 0.50), with a greater reduction than the AB group (difference-in-differences [DiD] = -107.3 ms; *p* = 0.019; d = 0.47). Short-term memory (Digit Span) improved only in the PAL group (Δ =  + 0.6; *p* < 0.001; d = 0.44), with larger gains than the CTL group (DiD =  + 0.7; *p* = 0.024; d = 0.54) and AB group (DiD =  + 0.7; *p* = 0.010; d = 0.49). Spatial reasoning (Mental Rotation) improved in both the PAL (Δ = -1967.5 ms; *p* < 0.001; d = 0.72) and AB groups (Δ = -1477.8 ms; *p* < 0.001; d = 0.54), but only the PAL group showed a greater change than the CTL group (DiD = -1394.0 ms; *p* = 0.012; d = 0.54). Spatial orientation (Posner Cueing) improved in all groups (PAL group: Δ = -386.6 ms; *p* < 0.001; d = 0.68; CTL group: Δ = -183.8 ms; *p* = 0.024; d = 0.29; AB group: Δ = -158.4 ms; *p* = 0.007; d = 0.36), with the PAL group presenting greater reductions than the CTL (DiD = -202.8 ms; *p* = 0.045; d = 0.33) and AB groups (DiD = -228.2 ms; *p* = 0.007; d = 0.45).

**Conclusions:**

Physically active lessons enhanced various cognitive functions, while active breaks, although less impactful, also represent a beneficial strategy.

**Trial registration:**

Brazilian Clinical Trials Registry (REBEC trial: RBR-10zxwdrh, retrospectively registered on 2025-01-09, https://ensaiosclinicos.gov.br/rg/RBR-10zxwdrh).

**Supplementary Information:**

The online version contains supplementary material available at 10.1186/s12966-025-01789-6.

## Background

The increase in the number of studies on sedentary behavior over the past few years reflects its recognition as a public health issue [1]. While the implications of this behavior are better understood in adults [2–6], there is growing evidence regarding the adverse effects of prolonged sitting in children, including obesity [7], elevated systolic and diastolic blood pressure [8], impaired vascular [9, 10] and cerebrovascular functions [11], impaired motor abilities [12, 13], and reduced sleep duration [14]. Such evidence has driven the implementation of policies and international recommendations to reduce sedentary behavior, particularly during childhood and adolescence [15–17], recognizing that lifestyle patterns established during these formative years tend to endure [18].

In this context, schools have been the focus of research and interventions aimed at reducing sedentary behavior [1], largely because of the significant amount of daily time that schoolchildren spend in this setting [19] and because, in more traditional teaching models, classroom layouts tend to encourage prolonged periods of sitting among students [19, 20]. Thus, strategies aimed at replacing sedentary behavior with physical activity and optimizing classroom time have been a focal point for researchers [21, 22]. The integration of physical activity in the classroom can occur in various forms [21], including active breaks [23] (i.e., short intervals during class for physical activities that may or may not be related to the curricular content) and physically active learning [24] (i.e., integrating physical activity into curricular content, such as teaching mathematics via body movement). These strategies have been suggested due to their low-cost nature [25, 26], and potential to reduce sedentary behavior [23, 24], increase physical activity levels [23, 24, 27], improve academic performance [24, 28], and yield promising results in cognitive outcomes such as inhibitory control [29–32], short-term memory [33–35], attention [34], and fluid intelligence [36].

Despite existing research, a gap remains in our understanding of how certain cognitive processes related to problem-solving and decision-making are affected. These processes encompass so-called executive functions [37], which control goal-directed behaviors, assist in impulse control, and help maintain a focus on tasks. These functions are particularly important because of their relationship with the development of the frontal cortex, a brain region involved in various higher-order cognitive functions (such as risk assessment, language production, visual processing, and short-term memory), which develops throughout childhood and adolescence [38]. The study of these functions could help clarify the evidence on cognitive outcomes, given that previous reviews [23, 24, 26, 39] have focused on a limited subset of executive functions, such as inhibitory control, short-term memory, and attention. Moreover, most research has been conducted in high-income countries, which may limit the generalizability of the findings to other educational contexts [23, 24]. For example, in Brazil, the existence of half-day schedules and poor infrastructure in schools, such as small classrooms and overcrowding [40], may pose critical limitations for the implementation of these interventions. Additionally, most studies have focused on a single type of intervention [23, 24, 26–28, 39], with few comparisons of the effects of different approaches [31, 41–44].

To the best of our knowledge, only two studies [45, 46] have compared interventions involving teacher-implemented physically active lessons and active breaks in children. However, both were conducted in high-income countries and focused on mathematical performance, without examining cognitive outcomes. Mavilidi & Vazou [45] evaluated an eight-week intervention in a non-randomized trial with 4th and 5th-grade students. The results indicated that physically active lessons had a more positive impact on mathematical performance than active breaks and traditional teaching. Conversely, Syväoja et al. [46] conducted a five-month cluster-randomized clinical trial with 3rd-grade students but found no significant differences in mathematical performance between the groups. Therefore, gaps remain in understanding the effects of these strategies, particularly regarding their impact on executive functions.

In addition, while these findings contribute to understanding the academic effects of such interventions, they leave an important gap regarding their impact on cognitive functions. This aspect is particularly relevant, as evidence suggests that executive functions are good predictors of academic achievement and overall success in school [47–49]. Furthermore, further research is needed to assess whether the hypothesized benefits extend to different educational contexts, such as low- and middle-income countries, in addition to studies comparing different types of interventions, particularly the differences between physically active lessons and active breaks. Therefore, the primary aim of the present study is to assess and compare the effects of physically active lessons and active breaks on the executive functions of elementary school students. The secondary objective is to examine the effects on physical activity levels, quality of life, daytime sleepiness, and school perception.

## Methods

The trial was reported following the CONSORT statement for cluster randomized trials [Supplementary Material 1] [50] and the TIDieR Checklist for Reporting and Replicating Interventions [Supplementary Material 2].

### Study design

**Fig. 1:**
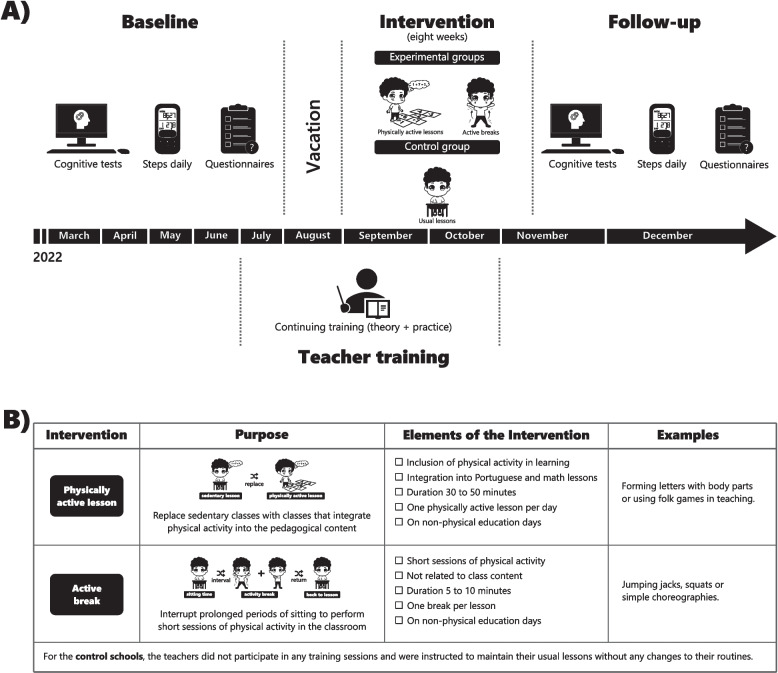
Schematic of the study design. **A** Schematic showing the study protocol timeline. **B** Description of the interventions

A three-arm cluster-randomized clinical trial was conducted in public schools in the city of Aracaju, Brazil. The data from this study are part of the first year of the Erguer project, conducted during the year 2022. The study received approval from the Human Research Ethics Committee of the Federal University of Sergipe (Approval Number: 5.301.398). Figure 1 illustrates the experimental design of the study, summarizing the main stages. Evaluations took place at two time points: the baseline assessment, conducted between March and July 2022, and a follow-up assessment after the interventions, conducted between November and December 2022. The interventions lasted eight weeks, starting in September, after the initial assessments and the return from vacation, and ended in November 2022. The interventions were implemented by the classroom teachers themselves, directly in their classrooms. Training and support for teachers were provided by the project team between July and October 2022, combining in-person and online sessions.

### Participant schools

One of the eight regions of the municipality of Aracaju [51] was randomly selected. All schools in this region, with at least two first-grade classes, were invited to participate. Six schools (100%) accepted the invitation and were randomly assigned to one of three groups: 1) an intervention group with physically active lessons (PAL, two schools); 2) an intervention group with active breaks (AB, two schools); and 3) a control group (CTL, two schools).

### Participant students

After primary consent was obtained from the school, all first-grade children were invited to participate in the study. The inclusion criteria required that children be regularly enrolled in the selected schools and return the informed consent form duly signed by their parents or guardians. Children with physical limitations (such as orthopedic problems, injuries, blindness, or debilitating chronic conditions) or cognitive/behavioral issues (such as hyperactivity or uncontrolled cognitive disorders) that would prevent participation in the planned activities or hinder comprehension of the assessments were excluded from the study.

### Sample size calculation

To estimate the required sample size for this three-arm repeated-measures design study, we used the “rmanova” function from the pwrss package in R. Initially, we defined a small-to-moderate expected effect size (partial η^2^) of 0.03 to detect a group-by-time interaction. We assumed a correlation between measurements of 0.50, an alpha level of 0.05, and a statistical power of 80%, which resulted in an ideal sample size of 81 participants. Next, we applied a design effect (DE) adjustment, calculated using the formula DE = 1 + (m – 1) × ρ, where we assumed a conservative intracluster correlation coefficient (ρ) of 0.05 based on previous studies [52], and average cluster size (m) of 15 students, based on a prior pilot study [30]. This yielded a design effect of 1.7 and an adjusted sample size of 138 participants.

Considering that only one of the eight educational regions was selected for the study, and each region had an average of six schools, we assumed the presence of only six possible clusters. We then recalculated the required cluster size to ensure that the adjusted sample of 138 students would retain the same statistical power as the originally estimated ideal sample of 81 participants. For this, we used the traditional formula for calculating the effective sample size (ESS), defined as ESS = (m × k)/DE, and rearranged as m = (ESS × DE)/k. This resulted in an estimated need to recruit an average of 23 participants per school. Finally, by incorporating an expected attrition rate of 20%, we arrived at a final recommended sample size of 166 participants.

### Training and intervention support

Teachers in the intervention schools were invited to participate in a 48-h training course, divided into four modules, provided by the project team. The sessions took place between July and October 2022, being conducted both in person and online, and tailored to the type of intervention (physically active lessons and active breaks). The training was structured into two modules before the interventions, covering both theoretical and practical sessions (concepts, definitions, organization, and planning), and two modules during the interventions (monitoring, discussing challenges, problem-solving, and sharing experiences). To support implementation, teachers were provided with educational materials, including ideas for activities, adapted lesson plans, and suggestions on how to modify the interventions to suit their specific contexts.

Additionally, an intern was assigned to each school. The interns were undergraduate students, and their primary role was to conduct assessments and, in the case of intervention schools, to offer support to teachers whenever possible. All the interns underwent both in-person and remote training sessions on data collection procedures.

### Intervention

All the students in the intervention classes participated in the activities, but only the students who were part of the sample were assessed. The interventions took place particularly on days without physical education classes (two days a week), as these are the days when children spend more time sitting during school. A typical school day in Aracaju (Brazil) runs from 7:00 am to 11:00 am, with a break from 9:00 am to 9:20 am for students who attend school only in the morning, and from 1:00 pm to 5:00 pm with a break from 3:00 pm to 3:20 pm for students who attend school only in the afternoon.

At the schools assigned to the PAL group, the teachers replaced sedentary lessons with lessons that integrated physical activity into the pedagogical content [53]. Adapted lesson plans were provided to the teachers, including strategies for incorporating movement into Portuguese language and math content (e.g., teaching content through folk games). The physically active lessons were designed to have the same duration as a typical daily lesson, ranging from 30–50 min. The teachers were instructed to initially conduct at least one physically active lesson per day, gradually increasing the frequency as they became more engaged and familiar with the interventions.

For the schools assigned to the AB group, the teachers were instructed to interrupt classroom activities or tasks after extended periods where the children remained seated (approximately 60 min). These breaks were designed to disrupt prolonged sitting by incorporating short sessions of moderate-intensity physical activity within the class. Each session lasted 5 to 10 min and followed a structure consisting of three phases: preparation, physical activity, and relaxation. In the preparation phase, which lasted between 1 and 4 min, the students stood next to their desks while the teacher explained the activity, with the duration varying based on the time needed to explain the task. The physical activity phase, lasting 2 to 3 min, included various exercises, such as aerobics (e.g., jumping jacks), strength and resistance exercises (e.g., squats), and playful activities, such as simple choreographies, mimicking movements, and active games. To reduce arousal after physical activity and redirect attention back to the previous task, cool-down exercises (e.g., stretches, breathing exercises) were performed during the final relaxation phase, which lasted 2–3 min. Teachers were instructed to implement at least one break per lesson or after 60 consecutive minutes of prolonged sitting (approximately two breaks per day), which gradually increased as they became more engaged and familiar with the interventions.

### Control

In schools assigned to the CTL group, the teachers did not participate in any training sessions and were instructed to maintain their usual lessons without any changes to their routines.

### Data collection procedures

Data collection took place at the schools and was conducted by the project interns. The children were taken out of the classroom one at a time and led to the assessment area. To minimize the time spent away from class, the tests were divided into two sessions of approximately 30 min. Therefore, children needed to return at least two times on different days during the data collection weeks to complete all the study assessments, with efforts made to schedule these sessions at the same time each day. Both the cognitive performance tests and questionnaires were administered on computers/laptops.

### Primary outcome assessment

#### Cognitive performance

Five executive functions were assessed: inhibitory control via the Go/NoGo paradigm [54]; short-term memory, via the nonverbal Digit Span forward test paradigm [55]; selective attention, via the visual search test paradigm [56]; spatial reasoning, via the Mental Rotation test paradigm [57]; and spatial orientation, via the Cueing Posner test [58]. For all the tests, computerized versions were used, programmed with a Psytoolkit [59, 60], and made available via JATOS [61]. The number of correct responses and reaction time (in milliseconds) for each test were used as indicators of cognitive performance. [for more details, see Additional File 3].

### Secondary outcomes assessed

#### Daily steps

For the objective assessment of physical activity, Omron HJA-310 pedometers were used for seven consecutive days, before and after the interventions. Participants were given information sheets detailing pedometer usage and customized belts for wearing the device on their waist. They were required to wear the belt with the pedometer continuously while awake, removing it solely for bathing and sleeping. Data were recorded on the total number of steps per day during a week (including weekdays and weekends) as well as the number of steps taken when the children arrived at school and before leaving (number of steps taken during school hours). For this study, valid information for total weekly steps was defined as at least two valid weekdays and one valid weekend day, a criterion based on common practice in the field and supported by a previous study in the same population group [62, 63]. Valid daily values range from a minimum of 1,000 steps to a maximum of 30,000 steps [64]. For steps at school, at least two days of use were needed, and to ensure that the device was used during school hours, daily values ​​of fewer than 100 steps were not considered valid. Finally, days on which participants reported not having worn the pedometer correctly throughout the day were also discarded.

#### Physical activity and screen-based activity

As a subjective measure, the daily frequency of physical activity and screen-based activity was assessed via the electronic School-Aged Children’s Dietary and Physical Activity Questionnaire (WEB-CAAFE), a web-based questionnaire where children use icons to report their previous day's physical activity, guided by an avatar. The child is instructed to recall the type, frequency, and intensity of the physical activities performed in the morning, afternoon, and evening of the previous day, selecting the respective icon [65]. The questionnaire was validated for administration only on days when the day preceding administration was not a weekend or a holiday. In other words, under the child’s normal routine, the questionnaire was administered from Tuesday through Friday. For example, the questionnaire administered on Friday referred to Thursday’s activities.

For this study, the daily frequency of physical activity was obtained by summing the reports of active play (i.e., play involving at least moderate physical activity, such as playing a ball), structured physical activity (i.e., physical activities monitored by a teacher or coach, such as swimming), and household chores (i.e., physical activities considered household tasks, such as sweeping). The daily frequency of screen-based activity was obtained by summing reports of screen use (smartphone, tablet, TV, laptop) across the three time periods (morning, afternoon, and evening). It is important to note that the questionnaire does not allow the participant to report the same activity more than once within a single period. For instance, if a participant reported playing in the park in both early and late morning, playing tag in the afternoon, and playing with a dog in the evening, their total count of active play would be 3, as the park icon could only be selected once in the morning. This restriction also applies to screen-based activities.

#### Quality of life

To assess the children's quality of life, the *Autoquestionnaire Qualité de Vie Enfant Imagé* (AUQEI) [66], validated for Brazilian children [67], was used. The questionnaire consists of 26 questions and generates a score ranging from zero to 78 points, with a higher score indicating a better perception of quality of life.

#### Daytime sleepiness

To evaluate daytime sleepiness, the Pediatric Daytime Sleepiness Scale (PDSS) [68], translated and validated into Brazilian Portuguese [69], was used. The questionnaire consists of eight questions and generates a score ranging from zero to 32 points, with a higher score indicating greater daytime sleepiness.

#### School perception

To assess students'perceptions of school, a questionnaire consisting of 15 questions was used: “*(1) How many siblings do you have? (2) Do you like school? (3) How much do you enjoy classroom activities/assignments? (4) How much do you like your teacher? (5) How do you feel at school? (6) Do you feel tired at school? (7) Do you have friends at school? (8) Do you play alone at school? (9) Do any children fight with you? (10) Do you fight with any children? (11) Do you complete your homework/assignments? (12) Do you receive help with your homework/assignments? (13) Who usually helps you? (14) Is there a place at home where you can study/do your homework? (15) What do you want to be when you grow up?”*. For the current study, questions (2), (3), and (4) were selected as they assess children's satisfaction with school, their teacher, and classroom assignments. Responses were given on a five-point visual analog scale, where facial expressions 1 and 2 were negative, 4 and 5 were positive, and 3 was neutral. For the analysis, responses were considered continuous values, where a higher score indicated greater satisfaction.

#### Demographics and anthropometrics

During baseline, information was collected on sex (male or female) and age (date of birth). Additionally, measurements of body weight (Seca® scale; accuracy of 0.1 kg) and height (accuracy of 0.1 cm) were taken via standardized procedures at each assessment point. Based on these measurements, body mass index (BMI) was calculated using the following equation: kg/m^2^. Additionally, BMI z-scores were calculated for each participant using the addWGSR() function from the R package *zscorer*, considering age and sex, as recommended by the World Health Organization [70].

### Statistical analyses

All analyses employed the intention-to-treat (ITT) method without data imputation, considering the information of all children regardless of missing data at any time point. The outliers were addressed via the winsorization technique [71], which prevents the permanent loss of extreme values by adjusting them to acceptable limits. A limit of three standard deviations was used for the minimum and maximum values.

Descriptive analyses are presented as relative and absolute frequencies, means, and standard deviations. Data normality was verified via the Shapiro–Wilk test. Baseline comparisons between groups were performed for descriptive and exploratory purposes using one-way ANOVA or the Kruskal–Wallis test for continuous variables, and Pearson's chi-square test for categorical variables. Generalized estimating equation (GEE) models with Gamma distributions (Poisson for count outcomes), and exchangeable working correlations were implemented via the glmgee() function from the *glmtoolsbox* package to assess the effects of interventions (physically active lessons, active break, and control) over time (baseline and follow-up) on the outcomes. Age, sex, and half-day school schedules were included as covariates in the analyses to control for potential confounding effects, as these variables are known to influence both physical activity levels and cognitive performance in children[72–74]. The selection of the GEE model was motivated by its various advantages: it does not require normally distributed data, it allows for the analysis of correlated observations [75, 76], and it is robust enough to handle missing data over time, if the data are Missing Completely at Random (MCAR) [77]. In our study, the missing data were related to changes in classes or participating schools, a process we consider random. To examine this issue more rigorously, we tested the MCAR hypothesis using the mcar_test() function from the *naniar* package, and the null hypothesis of MCAR was not rejected, supporting the adequacy of the GEE approach in our context. Furthermore, intracluster correlation coefficients (ICCs) of schools were reported, obtained from unadjusted follow-up raw scores via the clus.rho() function from the *FishMethods* package, adjusted for clusters with unequal sample sizes. For models that showed a significant group-by-time interaction, we conducted post-hoc comparisons using the contrast() function in *emmeans*, performing within-group pairwise tests of changes from baseline to follow-up, between-group comparisons at each time point (baseline and follow-up), and difference-in-differences contrasts of those changes.

Additionally, partial eta squared (η^2^) for the group-by-time interaction effect in the GEE models was obtained from the F statistics and degrees of freedom (effect and error) extracted by the joint_tests() function of the *emmeans* package and converted to partial η^2^ using Cohen's equation [78]. Cohen's d, as suggested by Becker [79], was calculated to determine the effect size for post hoc comparisons. All analyses were conducted via R version 4.4.0 within the RStudio integrated development environment. A significance level of *p* < 0.05 was adopted for all analyses.

## Results

### Baseline characteristics of the participants

**Fig. 2:**
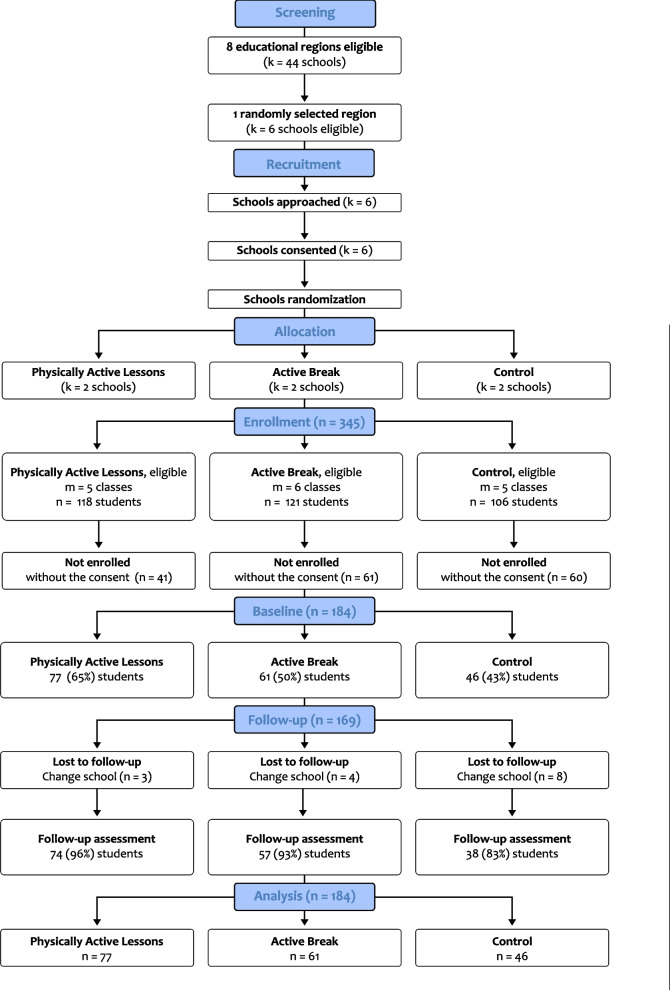
Flow diagram - CONSORT

The recruitment and data collection flowcharts are presented in Fig. 2. Out of the 345 first-grade children eligible across the six schools, 184 children (53.3%) returned a signed informed consent form from their parents. The physically active lessons (PAL) group included 77 children, the active break (AB) group included 61 children, and the control (CTL) group included 46 children. The characteristics of the children in the groups are presented in Table 1, with participants having an average age of 6.9 ± 0.6 years and 52.7% being girls. Differences between groups were observed at baseline for height (*p* < 0.001), half-day schedule (*p* < 0.001), mental rotation test (number of hits: *p* = 0.012; reaction time: *p* < 0.001), steps at school (*p* = 0.003), frequency of physical activity, and screen-based activity (*p* < 0.001). Further details are available in Supplementary Table 1 of Supplementary Material 4.

**Table 1:** Characteristics of participating children at baseline (*N* = 184)

	CTL (*n*=46)	PAL (*n*=77)	AB (*n*=61)	*p*	All
Demographic					
Age, years	7.2 ± 0.9	6.8 ± 0.4	6.9 ± 0.3	.106	6.9 ± 0.6
Sex (%)					
Boys	25 (54.3)	34 (44.2)	28 (45.9)	.530	87 (47.3)
Girls	21 (45.7)	43 (55.8)	33 (54.1)		97 (52.70
Anthropometric					
Height, m	1.21 ± 0.09	1.16 ± 0.07	1.16 ± 0.08	<.001	1.17 ± 0.08
Weight, kg	25.5 ± 6.6	23.8 ± 5.4	24.2 ± 6.3	.235	24.4 ± 6.0
BMI, Z score (WHO)	−0.07 ± 1.90	0.21 ± 1.89	0.28 ± 2.39	.697	0.16 ± 2.06
School additional information					
Half-day schedules (%)					
Morning	12 (26.1)	51 (66.2)	38 (62.3)	<.001	101 (54.9)
Afternoon	34 (73.9)	26 (33.8)	23 (37.7)		83 (45.1)

Characteristics are presented as the means ± standard deviations, except for sex and half-day schedules, which are presented as absolute and relative frequencies

Comparisons between groups at baseline were performed via one-way ANOVA or Kruskal‒Wallis test for continuous variables, depending on normality, and the chi‒square test for categorical variables

*p* <.05, statistically significant

*CTL* control, *PAL* physically active break, *AB* active break

### Main outcome

Significant interactions between group and time were observed in four out of the five tests conducted (Table 2). For the test Go/NoGo (reaction time: *p* = 0.045; partial η^2^ = 0.02), DigitSpan (correct responses: *p* = 0.020; partial η^2^ = 0.01), Mental Rotation (reaction time: *p* = 0.049; partial η^2^ = 0.02), and Cueing Posner (reaction time: *p* = 0.017; partial η^2^ = 0.03).

**Table 2:** Estimated marginal means for the primary outcomes of each group and results from the generalized estimating equation (GEE) models

Outcome			Estimated marginal means		GEE (group by time)		
		N	Baseline	Follow-up	*P*	Partial η^2^	ICC
Go/NoGo							
Correct responses (hits)^a,b^	CTL	44	23.7 (0.2)	24.3 (0.2)	.218	.01	.03
	PAL	77	23.1 (0.2)	23.9 (0.1)			
	AB	60	23.4 (0.2)	23.8 (0.2)			
Time reaction (milliseconds)^a,b^	CTL	44	913.4 (33.4)	875.2 (41.6)	.045	.02	.16
	PAL	77	833.9 (23.6)	729.5(22.7)			
	AB	60	886.1 (31.0)	886.2 (31.0)			
DigitSpan							
Correct responses (hits)	CTL	46	2.2 (0.2)	2.1 (0.2)	.020	.01	.07
	PAL	77	2.3 (0.2)	2.9 (0.2)			
	AB	59	2.3 (0.2)	2.2 (0.2)			
Mental Rotation							
Correct responses (hits)	CTL	44	12.7 (0.4)	12.0 (0.4)	.242	.01	<.001
	PAL	77	11.8 (0.3)	12.1 (0.2)			
	AB	60	12.1 (0.3)	12.4 (0.2)			
	CTL	44	5887.6 (365.2)	5314.1 (335.1)	.049		
Time reaction (milliseconds)^a,b^	PAL	77	7068.0 (310.6)	5100.5 (173.4)		.02	.03
	AB	60	7354.4 (353.2)	5876.6 (183.6)			
Cueing Posner							
Correct responses (hits)	CTL	39	23.6 (0.3)	23.5 (0.3)	.260	.01	.01
	PAL	77	23.3 (0.2)	23.9 (0.2)			
	AB	60	23.9 (0.2)	24.3 (0.2)			
Time reaction (milliseconds)^b^	CTL	39	1788.9 (102.8)	1606.1 (54.6)	.017	.03	.01
	PAL	77	1889.6 (64.1)	1503.2 (36.7)			
	AB	60	1655.8(56.6)	1497.8 (48.8)			
Visual Search							
Correct responses (hits)	CTL	44	6.5 (0.2)	6.7 (0.3)	.988	.00	.07
	PAL	77	6.8 (0.2)	6.9 (0.2)			
	AB	60	6.9 (0.2)	7.1 (0.2)			
Time reaction (milliseconds)^b^	CTL	44	3941.1 (201.9)	3137.6 (188.7)	.076	.02	.08
	PAL	77	3943.8 (132.1)	3088.2 (108.9)			
	AB	60	3932.5 (189.9)	3590.4 (140.0)			

*CTL* control, *PAL* physically active lesson, *AB* active break. The estimated marginal means and standard errors are based on generalized estimating equation (GEE) models and their respective adjustments. The GEE models were adjusted for age, sex and half-day schedules. The interpretation of the partial η^2^ effect size is as follows: small if < 0.06, medium if between 0.06 and 0.14, and large if ≥ 0.14

^a^statistically significant for the main effect of group

^b^statistically significant for the main effect of time

*p* <.05, statistically significant

**Fig. 3:**
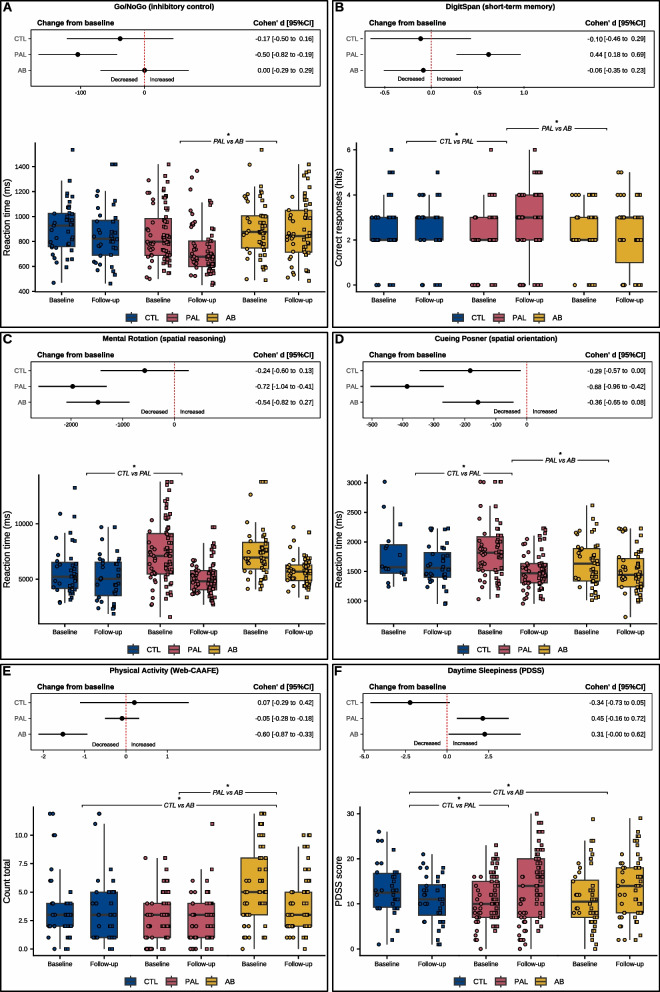
Boxplots and effect sizes for post hoc comparisons of within-group changes and between-group differences in change. The boxplots display the median and 25th and 75th percentiles of unadjusted scores (raw values), where"unadjusted"refers to the original data before any covariate corrections (e.g., age or sex), although the data were winsorized. Circles and squares represent individual children's results, identified by their respective schools. Point range indicates the changes from baseline (Δ) and the corresponding confidence intervals. Cohen’s d effect sizes were calculated based on GEE models and interpreted as follows: small (< 0.50), medium (0.50–0.80), and large (≥ 0.80). Brackets indicate statistically significant comparisons (*p* < 0.05) for differences in change between groups (difference-in-differences)

Figure 3 illustrates the variations in the outcomes that showed significant group-by-time interactions. The delta values (Δ, i.e., changes from baseline to follow-up) and the difference-in-differences (DiD, i.e., contrasts of mean changes between groups) are presented in Table 2 of Supplementary Material 4.

For inhibitory control (Fig. 3A), only the PAL group demonstrated a significant improvement in reaction time in the Go/No-Go test from baseline to follow-up (Δ = −106.4 ms; *p* < 0.001; d = 0.50). No significant changes were observed in the CTL group (Δ = −35.7 ms; *p* = 0.399; d = 0.17) or in the AB group (Δ = 0.8 ms; *p* = 0.981; d = 0.00). The change from baseline in the PAL group was significantly greater compared to the AB group (DiD = −107.3 ms; *p* = 0.019; d = 0.47), with a non-significant trend compared to the CTL group (DiD = −70.8 ms; *p* = 0.168; d = 0.33). At follow-up, the PAL group exhibited significantly faster reaction times than the CTL (mean difference at follow-up = −138.1 ms; *p* = 0.011) and AB groups (mean difference at follow-up = −159.9 ms; *p* < 0.001).

For short-term memory (Fig. 3B), a significant increase in the number of correct answers in the DigitSpan test was observed in the PAL group (Δ =  + 0.6; *p* < 0.001; d = 0.44). No significant change was observed in the CTL group (Δ = −0.1; *p* = 0.612; d = 0.10) or the AB group (Δ = −0.1; *p* = 0.705; d = 0.06). The difference in change from baseline in the PAL group was significantly greater compared to the CTL group (DiD =  + 0.7; *p* = 0.024; d = 0.54) and AB group (DiD =  + 0.7; *p* = 0.010; d = 0.49), while the AB group and CTL group did not differ (DiD =  + 0.1; *p* = 0.857; d = 0.04). At follow-up, the PAL group scored significantly higher than the CTL (mean difference at follow-up =  + 0.7; *p* = 0.038) and AB groups (mean difference at follow-up =  + 0.6; *p* = 0.021), with no difference between the AB group and CTL group (mean difference at follow-up =  + 0.0; *p* = 0.994).

For spatial reasoning (Fig. 3C), significant changes were identified from baseline in mental rotation test reaction times for the PAL group (Δ = −1967.5 ms; *p* < 0.001; d = 0.72) and AB group (Δ = −1477.8 ms; *p* < 0.001; d = 0.54). No significant change was observed in the CTL group (Δ = −573.5 ms; *p* = 0.187; d = 0.24). Compared to the CTL group, the PAL group showed a significantly greater reduction in reaction time (DiD = −1394.0 ms; *p* = 0.012; d = 0.54), while the AB group exhibited a non-significant trend in the same direction (DiD = −904.3 ms; *p* = 0.091; d = 0.35). At follow-up, the PAL group had significantly faster reaction times than the AB group (mean difference at follow-up = −776.1 ms; *p* = 0.006), with no significant differences between the PAL and CTL groups (mean difference at follow-up = −213.5 ms; *p* = 0.836) or between the AB and CTL groups (mean difference at follow-up = 562.5 ms; *p* = 0.298).

For spatial orientation (Fig. 3D), all groups showed significant reductions in reaction time from baseline on the Cueing Posner test: PAL group (Δ = −386.6 ms; *p* < 0.001; d = 0.68), CTL group (Δ = −183. ms; *p* = 0.024; d = 0.29) and AB group (Δ = −158.4 ms; *p* = 0.007; d = 0.36). The PAL group showed a significantly greater change compared to the CTL group (DiD = –202.8 ms; *p* = 0.045; d = 0.33) and the AB group (DiD = −228.2 ms; *p* = 0.007; d = 0.45), while the AB and CTL groups did not differ (DiD =  + 25.4 ms; *p* = 0.801; d = 0.05). At follow-up, reaction times were similar across groups (PAL group vs CTL group, mean difference at follow-up = −101.3 ms; *p* = 0.320; AB group vs CTL group, mean difference at follow-up = −106.9 ms; *p* = 0.360; PAL group vs AB group, mean difference at follow-up =  + 5.6 ms; *p* = 0.995).

### Secondary outcomes

Most secondary outcomes did not show statistical significance for the interaction between group and time (Table 3), except for the daily frequency of physical activity (*p* = 0.002; partial η^2^ = 0.04) and daytime sleepiness (*p* = 0.004; partial η^2^ = 0.04). Since the half-day schedules were significant in the models for these two outcomes (*p* < 0.001), we repeated the analyses by adding the interaction term (group × time × half-day) to examine any potential moderating effect of the half-day schedules. However, the interaction term was not significant [daily frequency of physical activity (*p* = 0.979); daytime sleepiness (*p* = 0.494)].

**Table 3:** Estimated marginal means for the secondary outcomes of each group and results from the generalized estimating equation (GEE) models

Outcome			Estimated marginal means		GEE (group by time)		
		*N*	Baseline	Follow-up	*P*	Partial η^2^	ICC
Steps							
At school (step/day)^a^	CTL	41	1724.5 (146.3)	1746.3 (148.4)	0.383	.01	.40
	PAL	73	1330.2 (105.9)	1137.4 (55.5)			
	AB	58	1767.4 (116.7)	1621.8 (110.4)			
In the week (step/day)^b^	CTL	40	7089.5 (455.0)	6244.7 (469.2)	0.564	.00	-.02
	PAL	61	6816.3 (320.5)	6605.3 (377.6)			
	AB	55	7044.9 (388.8)	6395.8 (399.9)			
Web-CAAFE (self-reported)							
Physical activity (frequency)^a^	CTL	46	3.8 (0.4)	4.0 (0.6)	0.002	0.04	.12
	PAL	77	2.4 (0.2)	2.3 (0.2)			
	AB	61	4.8 (0.3)	3.2 (0.3)			
Screen-based activity (frequency)^a^	CTL	46	1.3 (0.2)	1.9 (0.3)	0.071	0.02	.10
	PAL	77	2.1 (0.1)	1.8 (0.1)			
	AB	61	1.7 (0.1)	1.5 (0.2)			
Quality of life							
AUQEI Score (0–78 points)	CTL	45	56.2 (1.3)	53.4 (2.1)	0.129	0.01	.12
	PAL	77	53.7 (0.9)	54.0 (0.9)			
	AB	60	52.1 (1.1)	53.9 (1.2)			
Daytime Sleepiness							
PDSS Score (0–32 points)	CTL	43	14.0 (1.0)	11.8 (0.9)	0.004	0.04	.32
	PAL	77	9.9 (0.6)	12.0 (0.7)			
	AB	59	10.5 (1.0)	12.8 (0.9)			
School perception							
School (Likert Scale 1–5)^a, b^	CTL	43	4.8 (0.1)	4.8 (0.1)	0.082	0.02	.12
	PAL	77	4.8 (0.1)	4.7 (0.1)			
	AB	60	4.7 (0.1)	4.1 (0.2			
Teacher (Likert Scale 1–5)	CTL	43	4.5 (0.2)	4.6 (0.1)	0.095	0.01	-.02
	PAL	77	4.5 (0.1)	4.6 (0.1)			
	AB	60	4.8 (0.1)	4.6 (0.1)			
Task in the classroom (Likert Scale 1–5)^b^	CTL	43	4.3 (0.2)	4.1 (0.2)	0.124	0.01	.11
	PAL	77	4.4 (0.1)	4.4 (0.1)			
	AB	60	4.3 (0.2)	3.8 (0.2)			

*CTL* control, *PAL* physically active lesson, *AB* active break. The estimated marginal means and standard errors are based on generalized estimating equation (GEE) models and their respective adjustments. The GEE models were adjusted for age, sex and half-day schedules. The interpretation of the partial η^2^ effect size is as follows: small if < 0.06, medium if between 0.06 and 0.14, and large if ≥ 0.14

^a^statistically significant for the main effect of group

^b^statistically significant for the main effect of time

*p* <.05, statistically significant

For the daily frequency of physical activity (Fig. 3E), significant reductions from baseline were observed in self-reported frequency of physical activity for the AB group (Δ = −1.5; *p* < 0.001; d = 0.60). There was no significant change from baseline in the CTL group (Δ =  + 0.2; *p* = 0.763; d = 0.07) or PAL group (Δ = −0.1; *p* = 0.644; d = 0.05). The difference in change from baseline was greater in the AB group compared to the CTL (DiD = −1.7; *p* = 0.015; d = 0.62) and PAL groups (DiD = −1.4; *p* < 0.001; d = 0.64), with no significant difference between the PAL and CTL groups (DiD = −0.3; *p* = 0.663; d = 0.12). At follow-up, the PAL group remained lower than the CTL group (mean difference at follow-up = −1.7; *p* = 0.022), the AB group did not differ from the CTL group (mean difference at follow-up = −0.8; *p* = 0.464), and the AB group continued to report a higher frequency of physical activity than the PAL group (mean difference at follow-up =  + 0.9; *p* = 0.013).

For daytime sleepiness (Fig. 3F), significant changes from baseline were observed, with increased daytime sleepiness in the PAL (Δ =  + 2.2; *p* = 0.007; d = 0.45) and AB groups (Δ =  + 2.3; *p* = 0.040; d = 0.31), while the CTL group did not show any change (Δ = −2.2; *p* = 0.068; d = 0.34). The difference in change from baseline was significantly greater in the PAL group compared to the CTL group (DiD =  + 4.4; *p* = 0.002; d = 0.76) and in the AB group compared to the CTL group (DiD =  + 4.5; *p* = 0.006; d = 0.65). There was no significant difference between the AB group and PAL group (DiD =  + 0.1; *p* = 0.928; d = 0.02). At follow-up, there were no significant group differences (PAL vs. CTL: mean difference =  + 0.3; *p* = 0.969; AB vs. CTL: mean difference =  + 1.0; *p* = 0.691; AB vs. PAL: mean difference = −0.8; *p* = 0.762).

## Discussion

In the current study, our primary objective was to investigate the effects of physically active lessons and active breaks on executive functions and health indicators in elementary school children. To the best of our knowledge, this is the first study to examine the effects of these two different strategies of school-based physical activity interventions on the executive functions of first-grade children from a low socioeconomic status region in a middle-income country.

The results of the cognitive tests revealed positive effects of physically active lessons on various executive functions. Regarding inhibitory control, as measured by the Go/NoGo test, a significant reduction in reaction time was observed in the PAL group. Furthermore, between-group comparisons of changes from baseline to follow-up showed that this group presented greater reductions in reaction times than the AB group, with a similar but non-significant trend relative to the CTL group. These findings suggest an improvement in the ability to inhibit impulsive responses, as reflected in faster reaction times at follow-up compared to both the CTL and AB groups. In terms of short-term memory, as assessed by the DigitSpan test, the PAL group demonstrated the greatest improvement in correct responses from baseline to follow-up, outperforming both the CTL and AB groups. These findings suggest an enhanced capacity to retain and mentally manipulate information resulting from participation in physically active lessons. Inhibitory control and short-term memory are cognitive functions associated with the prefrontal cortex [37]. The importance of these executive functions for academic performance is well established, as these skills are essential for complex tasks, problem solving, and effective learning, and underpin many other cognitive processes [80]. Our findings align with evidence reported in the literature, with recent studies identifying positive effects of physically active lessons on cognitive functions such as inhibitory control [29, 30] and short-term memory [33, 34]. Notably, the studies by Barboza et al. [29] and Oliveira et al. [30] are part of a pioneering pilot study, in which a clinical trial was conducted in a school in a city in the Northeast of Brazil, where physically active lessons were implemented for children in the 2nd grade of elementary school. Thus, our findings expand knowledge in the area by including a larger sample and expanding the number of schools evaluated.

For spatial reasoning, as measured by the mental rotation task, both the PAL and AB groups showed reductions in reaction time from baseline to follow-up, with a larger effect observed in the PAL group. Compared to the CTL group, only the PAL group demonstrated a significantly greater improvement, while the AB group showed a non-significant trend in the same direction. Spatial reasoning plays a crucial role in learning disciplines such as science, technology, engineering, arts, and mathematics, as it involves skills in visualizing and manipulating shapes and spaces, which are essential for understanding these concepts [81]. The CTL group, which showed better initial performance compared to the other groups, did not exhibit improvements during the study. This suggests that, for children with higher initial performance levels, the absence of an intervention may limit further development. In contrast, the initial differences between the intervention and control groups disappeared at follow-up, indicating that the interventions contributed to reducing early disparities in visuospatial processing. These findings suggest that strategies integrating physical activity into the classroom may be effective in enhancing skills such as spatial reasoning.

Finally, in spatial orientation, as measured by the Posner Cueing Test, all groups showed significant improvements from baseline, with the largest reduction in reaction time observed in the PAL group. Furthermore, improvement in the PAL group was significantly greater than in both the CTL and AB groups. These findings suggest that integrating physical activity into academic lessons may lead to more substantial gains in spatial orientation compared to other approaches. The Cueing Posner test evaluates the ability to voluntarily and quickly direct attention to a specific location in space based on visual cues [82, 83]. This skill is crucial for learning, as it allows students to focus their attention on relevant information and ignore distractions, thereby facilitating efficient and effective information processing.

One of the mechanisms that explains how physical activity can improve cognitive functions is the increase in blood flow to the prefrontal cortex during moderate to vigorous physical activity. This increased blood flow can trigger the release of various neurochemicals, such as growth factors and neurotransmitters, increasing the activity in this brain area [38, 84, 85]. This increase in brain activity is associated with improvements in executive functions [86]. Additionally, studies on isotemporal substitutions suggest that replacing prolonged sitting with light activity may also yield cognitive benefits [87], particularly when considering not only the intensity of activity but also the frequency with which sedentary time is interrupted [39]. The complexity of the approaches adopted can range from activities solely focused on movement and heart rate elevation (e.g., aerobic exercises) to more structured interventions involving greater cognitive engagement (such as dual-task exercises or those imposing more demanding cognitive challenges), as well as activities integrated into the school curriculum (e.g., solving mathematical problems while engaging in physical movement) [23, 25, 39]. Furthermore, the effectiveness of the intervention may also be linked to socioemotional factors, such as enjoyment of the activity, stress reduction, and socialization among peers [88].

In line with this perspective, the implementation of a more dynamic and playful classroom structure, where students spend less time sitting and more time engaged in light physical activities (such as standing or walking), may help explain the positive results observed in the group that participated in physically active lessons. In these lessons, movement is integrated into one or more partial learning processes of a specific knowledge or skill [53]. Moreover, physically active lessons often involve restructuring the classroom environment, such as rearranging desks and chairs, making it more dynamic and interactive. This factor may have contributed to increased engagement and attention to tasks [89], as well as promoting socialization and stress reduction, and making the lessons more enjoyable. Simultaneously activating multiple brain areas during learning through movement may favor information retention and effective learning [90]. On the other hand, the active breaks in this study focused primarily on interrupting prolonged sitting time, without involving pedagogical content or cognitive engagement. Consequently, the results for cognitive functions were less pronounced. A possible explanation seems to be related to the type of activity performed, as activities with more cognitively engaging components or those combined with the curriculum [25], have been shown to facilitate improvements in cognitive functions compared with active breaks with purely physical activity components [23].

Unexpectedly, a significant reduction was observed in self-reported daily frequency of physical activity only in the active breaks group. Although this group reported a higher daily frequency of physical activity at baseline compared to the other groups, the decline was substantial. Furthermore, the reduction was significantly greater than that observed in both the CTL and PAL groups. These findings indicate that, despite initially higher activity levels, the active breaks group experienced a marked decline over time that was not present in the other groups. One possible explanation for this finding may be related to the instrument used for assessment. While some traditional instruments measure physical activity based on duration and frequency to calculate total activity time, the Web-CAAFE adopts a different approach, measuring the frequency with which various activities are performed in blocks of periods (morning, afternoon, and evening). For instance, a participant who reported engaging in park play and riding a bike in the morning, tag in the afternoon, and playing with a dog in the evening would receive a total active play count of four. It is important to highlight that the same activity conducted within the same period is only counted once. Thus, if children engaged in the same activity (e.g., playing soccer) several times during the morning, the questionnaire considers that activity only once for that period. On the other hand, no significant differences were found in the average daily steps during the week or school hours. These results may indicate that, although the interventions were designed to create more dynamic classes, this was not reflected in the step count. A possible explanation, particularly for the lack of differences in steps during school time, is that assessments were conducted at baseline and after the intervention period. Although teachers were not instructed to discontinue the activities, it is possible that not all of them maintained the intervention consistently until the follow-up data collection, indicating a discontinuity in implementation. Future studies should consider conducting assessments during the intervention period itself and employing device-based measures of physical activity, such as accelerometers and inclinometers, to obtain more accurate estimates of movement.

Regarding daytime sleepiness, both the PAL and AB groups showed significant increases from baseline, while no significant change was observed in the CTL group. These increases were unexpected and may suggest that factors beyond the intervention itself influenced children's sleepiness levels. Importantly, the increases in sleepiness were significantly greater in both intervention groups compared to the CTL group. Although no group differences were detected at follow-up, these findings highlight the need for further investigation of potential contributors, such as sleep quality or scheduling, that may have impacted students’ daytime alertness. The half-day schedules did not moderate the observed effects, however, they did explain part of the observed changes. Information such as sleep duration, use of electronics before bedtime, and bed and wake-up times, among others, would help us better understand these changes. Previous evidence has associated daytime sleepiness with the number of hours of sleep [91] and the use of electronics before bedtime [92]. Moreover, bed and wake-up times can provide insights into the circadian cycle, given that disturbances in this cycle have also been linked to daytime sleepiness [93].

### Limitations and strengths

The current study presents some limitations that should be considered. First, to allow for greater external validity of the interventions, some decisions were made to ensure implementation primarily within the educational reality of each school. For example, teachers were instructed to implement the interventions as frequently as possible autonomously. Unlike other studies where teachers are directed to include a fixed number of interventions, in the present study, teachers had the freedom to schedule the activities as they wished. The implementation of the interventions gradually increased in frequency over the weeks. However, as there was no daily control of how often the activities were implemented, it is possible that the activities were conducted at various frequencies and times. We also note that some uncollected information, such as sitting time, hours of sleep, and socioeconomic information, could provide additional context of the participants.

On the other hand, the study has strengths, such as being a cluster-randomized clinical trial, which allows for more robust inferences about causality. Additionally, this was the first study to compare the effectiveness of two physical activity interventions in the classroom in a middle-income country in South America, thus expanding the knowledge in this area. Cognitive functions were assessed via standardized tests, which increases the reliability of the results and allows comparisons with other studies. Furthermore, information was collected on behaviors outside the school context, providing a comprehensive view of the reach of the interventions.

### Practical implications

This study demonstrates the effectiveness of incorporating increased movement into the classroom to enhance cognitive functions, a particularly relevant aspect within the educational context of a middle-income country such as Brazil. Given that many schools operate on a half-day basis and face multiple interrelated challenges, such as limited classroom sizes, overcrowding, excessive teacher workload, and low salaries, these factors collectively hinder the implementation of new strategies. Furthermore, to ensure positive results, the proposed activities must go beyond the mere inclusion of physical activity and integrate dynamic and engaging elements, as in the case of physical activity classes. Consequently, these findings underscore the necessity for a paradigm shift in educational practice, advocating for methods that foster a synergistic relationship between movement and learning, and ultimately contributing to a more holistic development of children's skills.

## Conclusion

We conclude that physically active lessons can improve inhibitory control, short-term memory, spatial orientation, and spatial reasoning in elementary school children. While less effective, active breaks still represent a viable strategy with no harmful effects. However, further research is needed on the long-term effects in order to determine best practices for implementing these interventions in schools.

## Supplementary Information

Supplementary Material 1.

Supplementary Material 2.

Supplementary Material 3.

Supplementary Material 4.

## Data Availability

The datasets used and/or analyzed during the current study are available from the corresponding author on reasonable request.

## Abbreviations

PAL: Physically active lessons
AB: Active Break
CTL: Control
ESS: Effective sample size
DE: Design effect
ICC: Intracluster correlation coefficient
DFPA: Daily frequency of physical activity
DFSA: Daily frequency of screen-based activity
AUQEI: Autoquestionnaire Qualité de Vie Enfant Imagé
PDSS: Pediatric Daytime Sleepiness Scale
BMI: Body mass index
ITT: Intention-to-treat
GEE: Generalized estimating equation
MCAR: Missing Completely at Random
DiD: Difference-in-Differences
