# Supplementary material for: Effects of physically active lessons and active breaks on cognitive performance and health indicators in elementary school children: a cluster randomized trial
Source: Int J Behav Nutr Phys Act. 2025 Jul 9;22:96. doi: 10.1186/s12966-025-01789-6 (PMC12243236; doi:10.1186/s12966-025-01789-6)
Supplement: Supplementary file 3 — Supplementary Material 3. [file 12966_2025_1789_MOESM3_ESM.docx]

**DESCRIPTION OF COGNITIVE TESTS**

The main outcome included the assessment of five constructs of cognitive function: inhibitory control, selective attention, spatial reasoning, spatial orientation, and short-term memory. In all cases, computerized versions of the tests were used, programmed with Psytoolkit [1,2], and made available via JATOS [3]. The number of correct responses and reaction time (in milliseconds) for each test were used as indicators of cognitive performance. The tests were conducted during the school period in which the child was studying and administered by project interns trained to administer the tests. Children were individually escorted from the classroom to the evaluation room with the evaluator. The total number of correct answers and the time taken (in milliseconds) to complete each test were used as indicators of cognitive performance.

**Supplementary Table 1.** Illustration and description of the cognitive test.

| **Cognitive test** | **Description** |
| --- | --- |
| 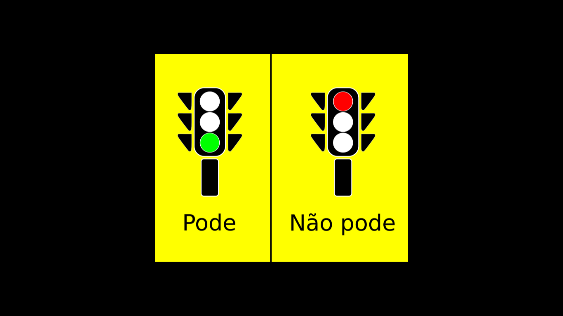 | Inhibitory control was assessed using the Go/NoGo paradigm [4]. The test consisted of 25 trials in which a traffic-light figure alternated randomly between green and red. When the green traffic light appeared, the child was required to press a computer key. When the red traffic light appeared, the child was required to wait for the figure to change without pressing the key. |
| **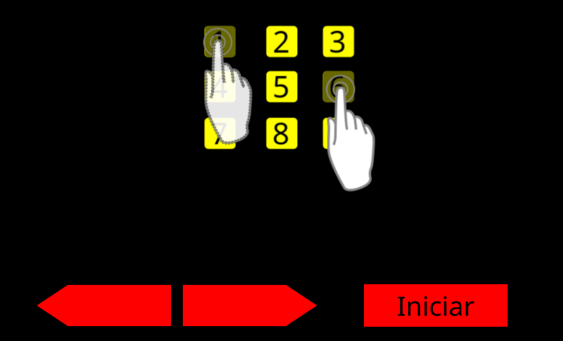** | Short-term memory was assessed using the Digit Span Forward test [5]. The test involved the presentation of a sequence of digits, and the child needed to repeat the presented sequence. If the child was able to repeat the sequence, a longer sequence of digits would be presented. The length of the digit sequence only increased after the participant correctly repeated the same sequence at least twice. The test ended when the child was no longer able to repeat the sequence of numbers for two consecutive trials. |
| 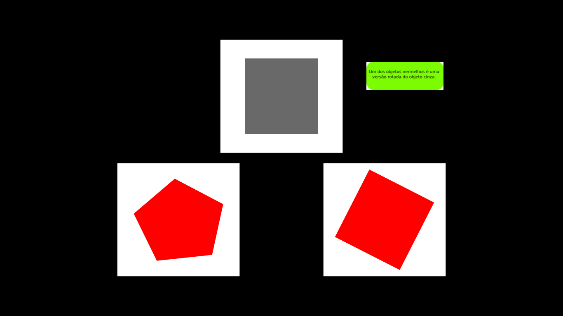 | Spatial reasoning was assessed using the Mental Rotation test [6]. The test consisted of 15 trials in which participants were presented with two rotated alternatives and had to select the one matching the gray example figure (rotated either right or left). The child was instructed to press the corresponding keys (alternative 1 or 2) to respond. |
| 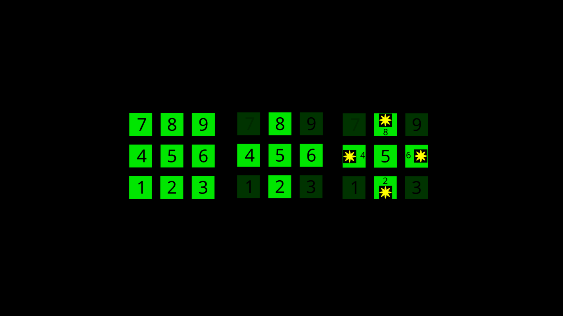 | Spatial orientation was also assessed using the Posner Cueing paradigm [7]. The test comprised 25 trials, each presenting four squares arranged in a cross (top, bottom, right, and left). In each trial, a valid cue (an asterisk) appeared in one square, and the child was instructed to press the number corresponding to that square on the numeric keypad, while ignoring an invalid cue (an arrow). On some trials, the arrow pointed to a different location than the asterisk to “trick” the child into making an error. |
| 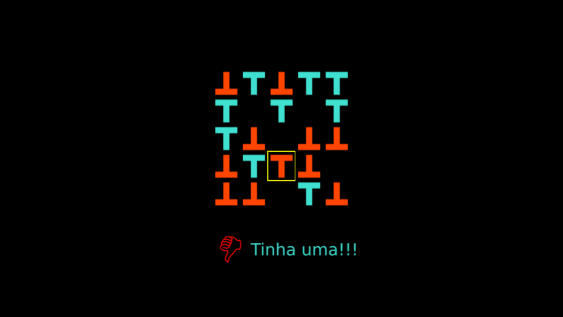 | Attention selective was assessed using the Visual Search test paradigm [8]. The test involved 15 trials in which figures containing multiple randomly distributed “T” letters were presented. The child was instructed to press a key on the computer each time the letter a red T appeared in its normal (non-inverted) position, even if there was only one such letter among many others. If the figure displayed the letter T in an inverted position or in another color (blue), the child was to wait for the figure to change without pressing the key. |

References

1. PsyToolkit: A Novel Web-Based Method for Running Online Questionnaires and Reaction-Time Experiments - Gijsbert Stoet, 2017 [Internet]. [cited 2024 Aug 29]. Available from: https://journals.sagepub.com/doi/abs/10.1177/0098628316677643

2. Stoet G. PsyToolkit: A software package for programming psychological experiments using Linux. Behav Res Methods [Internet]. 2010 [cited 2024 Aug 29];42:1096–104. Available from: https://doi.org/10.3758/BRM.42.4.1096

3. Lange K, Kühn S, Filevich E. "Just Another Tool for Online Studies” (JATOS): An Easy Solution for Setup and Management of Web Servers Supporting Online Studies. PLOS ONE [Internet]. 2015 [cited 2024 Jul 29];10:e0130834. Available from: https://journals.plos.org/plosone/article?id=10.1371/journal.pone.0130834

4. Criaud M, Boulinguez P. Have we been asking the right questions when assessing response inhibition in go/no-go tasks with fMRI? A meta-analysis and critical review. Neurosci Biobehav Rev. 2013;37:11–23.

5. Jones G, Macken B. Questioning short-term memory and its measurement: Why digit span measures long-term associative learning. Cognition. 2015;144:1–13.

6. Jost L, Jansen P. Manual training of mental rotation performance: Visual representation of rotating figures is the main driver for improvements. Q J Exp Psychol 2006 [Internet]. 2022 [cited 2024 Feb 22];75:695–711. Available from: https://www.ncbi.nlm.nih.gov/pmc/articles/PMC8915228/

7. Hayward DA, Ristic J. Measuring attention using the Posner cuing paradigm: the role of across and within trial target probabilities. Front Hum Neurosci [Internet]. 2013 [cited 2024 Feb 22];7:205. Available from: https://www.ncbi.nlm.nih.gov/pmc/articles/PMC3656349/

8. Treisman A. Focused attention in the perception and retrieval of multidimensional stimuli. Percept Psychophys [Internet]. 1977 [cited 2024 Feb 22];22:1–11. Available from: https://doi.org/10.3758/BF03206074
