# Supplementary material for: Effects of physically active lessons and active breaks on cognitive performance and health indicators in elementary school children: a cluster randomized trial
Source: Int J Behav Nutr Phys Act. 2025 Jul 9;22:96. doi: 10.1186/s12966-025-01789-6 (PMC12243236; doi:10.1186/s12966-025-01789-6)
Supplement: Supplementary file 4 — Supplementary Material 4. [file 12966_2025_1789_MOESM4_ESM.docx]

**Suplementary table 1.** Baseline comparisons between groups (N = 184).

|  | **CTL** | **PAL** | **AB** |  | **All** |
| --- | --- | --- | --- | --- | --- |
|  | Median (IQR) | Median (IQR) | Median (IQR) | p | Median (IQR) |
| **Cognitive test** |  |  |  |  |  |
| Go/NoGo |  |  |  |  |  |
| Correct responses | 24 (2) | 23 (2) | 24 (2) | .059 | 24 (3) |
| Time reaction | 927 (267) | 797 (296) | 876 (257) | .233 | 850 (297) |
| DigitSpan |  |  |  |  |  |
| Correct responses | 2 (1) | 2 (1) | 2 (1) | .891 | 2 (1) |
| Cueing Posner |  |  |  |  |  |
| Correct responses | 24 (2) | 24 (3) | 24 (2) | .235 | 24 (2) |
| Time reaction | 1569 (481) | 1818 (573) | 1632 (548) | .068 | 1738 (593) |
| Visual Search |  |  |  |  |  |
| Correct responses | 7 (1) | 7 (2) | 7 (2) | .514 | 7 (2) |
| Time reaction | 3801 (1530) | 3895 (1796) | 3971 (1473) | .857 | 3882 (1701) |
| Mental Rotation |  |  |  |  |  |
| Correct responses | 14 (3) a | 12 (3) ab | 12 (2) b | **.012** | 12 (3) |
| Time reaction | 5227 (2349) a | 7104 (3655) b | 6947 (2395) b | **< .001** | 6563 (3356) |
| **Movement behaviors** |  |  |  |  |  |
| Pedometer data (Steps/day) |  |  |  |  |  |
| At school | 1640 (1202) a | 1202 (875) b | 1589 (1143) a | **.003** | 1412 (1230) |
| In the week | 6633 (3031) | 5962 (2944) | 6801 (3925) | .850 | 6435 (3301) |
| Web-CAAFE |  |  |  |  |  |
| Physical activity | 3 (2) a | 3 (3) a | 5 (5) b | **< . 001** | 3 (3) |
| Screen-based activity | 1 (2) a | 2 (1) b | 2 (1) a | **< .001** | 2 (2) |
| **Health indicators** |  |  |  |  |  |
| Quality of life |  |  |  |  |  |
| AUQEI score | 55 (10) | 54 (9) | 52 (11) | .407 | 54 (10) |
| Daytime Sleepiness |  |  |  |  |  |
| PDSS score | 13 (8) | 10 (8) | 11 (8) | .098 | 11 (8) |
| **School perception** |  |  |  |  |  |
| Like school | 5 (0) | 5 (0) | 5 (0) | .580 | 5 (0) |
| Like teacher | 5 (0) | 5 (1) | 5 (0) | .093 | 5 (1) |
| Like task in the classroom | 5 (1) | 4 (1) | 5 (1) | .950 | 5 (1) |

**Note.** CTL, control; PAL, physically active break; AB, active break. Values are presented as median and interquartile range for unadjusted baseline results. The unadjusted results refer to the raw data prior to any adjustments for covariates (such as age or sex), but the data have undergone winsorization. One-way ANOVA was used for comparisons between groups at baseline for normally distributed variables, and the Kruskal-Wallis test was used for non-normally distributed variables. Different letters indicate significant differences between groups, with different letters signifying significant differences (e.g., Group 1: a; Group 2: b; Group 3: b indicates that Groups 2 and 3 are not different from each other but are different from Group 1).

**Suplementary table 2.** Changes from baseline to follow-up within groups and differences-in-differences between groups for cognitive performance, frequency of physical activity and daytime sleepiness.

|  | **Changes from baseline to follow-up** | | | | |  | **Contrasts of the changes between groups** | | | | | | | |
| --- | --- | --- | --- | --- | --- | --- | --- | --- | --- | --- | --- | --- | --- | --- |
|  | CTL |  | PAL |  | AB |  | PAL vs CTL |  |  | AB vs CTL |  |  | PAL vs AB |  |
|  | Δ [95%CI] |  | Δ [95%CI] |  | Δ [95%CI] |  | DiD [95%CI] | p |  | DiD [95%CI] | p |  | DiD [95%CI] | p |
| **Go/NoGo**  Time reaction  (milliseconds) | –35,7  [–118,8; 47,5] |  | **–106,4***  **[–168,2; –44,7]** |  | 0,8  [–68,9; 70,6] |  | –70,8  [–171,5; 29,9] | .168 |  | 36,5  [–69,2; 142,2] | .497 |  | -107,3  [-196.9; -17,6] | **.019** |
|  | Cohen’s d = .17 |  | Cohen’s d = .50 |  | Cohen’s d = .00 |  | Cohen’s d = .33 |  |  | Cohen’s d = .16 |  |  | Cohen’s d = .47 |  |
| **DigitSpan**  Correct responses  (hits) | –0,1  [–0,7; 0,4] |  | **0,6***  **[0,2; 0,9]** |  | –0,1  [–0,5; 0,3] |  | 0,7  [0,1; 1,4] | **.024** |  | 0,1  [–0,6; 0,7] | .857 |  | 0,7  [0,1; 1,2] | **.010** |
|  | Cohen’s d = .10 |  | Cohen’s d = .44 |  | Cohen’s d = 0.06 |  | Cohen’s d = .54 |  |  | Cohen’s d = .04 |  |  | Cohen’s d = .49 |  |
| **Mental Rotation**  Time reaction  (milliseconds) | –573.5  [–1426.0; 279.1] |  | **–1967.5***  **[–2627.5; –1307.5]** |  | **–1477.8***  **[–2087.3; –868.3]** |  | –1394,0  [–2476; –312] | **.012** |  | –904.3  [–1955; 146] | .091 |  | -490,0  [–1363.0; 384.0] | .270 |
|  | Cohen’s d = .24 |  | Cohen’s d = .72 |  | Cohen’s d = .54 |  | Cohen’s d = .54 |  |  | Cohen’s d = .35 |  |  | Cohen’s d = .18 |  |
| **Cueing Posner**  Time reaction  (milliseconds) | **–183.8***  **[–343,0; –24,3]** |  | **–386.6***  **[–505,0; –268,4]** |  | **–158,4***  **[–273,0; –44,3]** |  | –202.8  [–401,0; –4,5] | **.045** |  | 25,4  [–173,2; 224,1] | .801 |  | -228,2  [-392.7; -63,7] | **.006** |
|  | Cohen’s d = .29 |  | Cohen’s d = .68 |  | Cohen’s d = .36 |  | Cohen’s d = .33 |  |  | Cohen’s d = |  |  | Cohen’s d = .45 |  |
| **Web-CAAFE**  Physical activity  (frequency) | 0,2  [–1,1; 1,5] |  | –0,1  [–0,5; 0,3] |  | **–1,5***  **[–2,1; –0,9]** |  | –0,3  [–1,6; 1,0] | .663 |  | –1,7  [–3,1; –0,3] | **.015** |  | –1,4  [–2,1; –0,7] | **<.001** |
|  | Cohen’s d = .07 |  | Cohen’s d = .05 |  | Cohen’s d = .60 |  | Cohen’s d = .12 |  |  | Cohen’s d = .62 |  |  | Cohen’s d = .64 |  |
| **Daytime Sleepiness** PDSS score  (0 – 32 points) | –2,2  [–4,6; 0,1] |  | **2,2***  **[0,5; 3,7]** |  | **2,3***  **[0,1; 4,4]** |  | 4,4  [1,6; 7,1] | **.002** |  | 4,5  [1,3; 7,7] | **.006** |  | -0,1  [–2,7; 2,5] | .928 |
|  | Cohen’s d = .34 |  | Cohen’s d = .45 |  | Cohen’s d = .31 |  | Cohen’s d = .76 |  |  | Cohen’s d = .65 |  |  | Cohen’s d = .02 |  |

**Note.** CTL, control; PAL, physically active break; AB, active break. Contrasts are presented as changes from baseline (∆) or difference-in-differences (DiD) with respective confidence intervals (95% CI). Marginal estimated means are based on generalized estimating equation models. *, statistically significant for change from baseline.
